# Genome-Wide Association Studies (GWAS) and Transcriptome Analysis Reveal Male Heterogametic Sex-Determining Regions and Candidate Genes in Northern Snakeheads (*Channa argus*)

**DOI:** 10.3390/ijms252010889

**Published:** 2024-10-10

**Authors:** Haiyang Liu, Jin Zhang, Tongxin Cui, Weiwei Xia, Qing Luo, Shuzhan Fei, Xinping Zhu, Kunci Chen, Jian Zhao, Mi Ou

**Affiliations:** 1 Key Laboratory of Tropical and Subtropical Fishery Resources Application and Cultivation, Ministry of Agriculture and Rural Affairs, Pearl River Fisheries Research Institute, Chinese Academy of Fishery Sciences, Guangzhou 510380, China; hyliu@prfri.ac.cn (H.L.); pethap@outlook.com (J.Z.); ctx220098@163.com (T.C.); xiaweiwei727@163.com (W.X.); luoqing@prfri.ac.cn (Q.L.); szfei1994@163.com (S.F.); zhuxinping_1964@163.com (X.Z.); chenkunci@prfri.ac.cn (K.C.); 2 College of Fisheries and Life Sciences, Shanghai Ocean University, Shanghai 201306, China

**Keywords:** northern snakehead, GWAS, Fst, sex markers, candidate genes

## Abstract

The Northern snakehead (*Channa argus*) is a significant economic aquaculture species in China. Exhibiting sexual dimorphism in the growth rate between females and males, mono-sex breeding holds substantial value for aquaculture. This study employed GWAS and transcriptome analysis were applied to identify sex determination genomic regions and develop sex-specific markers. A total of 270 single-nucleotide polymorphisms (SNPs) and 31 insertion-deletions (InDels) were identified as being sexually dimorphic through GWAS and fixation index (Fst) scanning. Based on GWAS results, two sex-specific InDel markers were developed, effectively distinguishing genetic sex for XX females, XY males, and YY super-males via (polymerase chain reaction) PCR amplification. A major genomic segment of approximately 115 kb on chromosome 3 (Chr 03) was identified as the sex-determination region. A comparative transcriptome analysis of gonads for three sexes identified 158 overlapping differentially expressed genes (DEGs). Additionally, three sex-related candidate genes were identified near the sex determination region, including *id2*, *sox11*, and *rnf144a*. Further studies are required to elucidate the functions of these genes. Overall, two sex-specific InDel markers support a male heterogametic XX/XY sex-determination system in Northern snakeheads and three candidate genes offer new insights into sex determination and the evolution of sex chromosomes in teleost fish.

## 1. Introduction

The diversity and plasticity of sex determination systems in teleosts represent a fascinating area of study with profound implications for aquaculture, evolutionary biology, and conservation. With their range of sex chromosomes, sex-determining genes, and mechanisms of sex differentiation, teleosts offer unique insights into how sex determination systems evolve and adapt to various environmental pressures [1,2]. Fish gonadal development encompasses a series of intricate biological processes, including sex determination and differentiation. Fish sex determination can generally be classified into three types: genotypic sex determination (GSD), environmental sex determination (ESD), or a combination of both (GSD + ESD) [3]. Gonadal development initiates with sex determination. Through the influence of sex-determining genes, the genetic switch that triggers ovarian and testicular development is activated, subsequently inducing the downstream genes related to sex differentiation to regulate the expression of sex steroid synthase genes (ultimately determining the fate of the gonads), and their sex-determining genes remain unclear, making it challenging to fully understand the genetic basis of sex determination. However, advances in genomic analyses are helping to unravel the complex and plastic systems of sex determination in fish [4]. With the development of genomic tools combined with the application of quantitative genetics, more and more fish sex determination types and sex chromosomes have been demonstrated [4]. Fish species exhibit a wide range of sex determination modes, from simple genetic systems like XX/XY and ZZ/ZW to more complex systems involving multiple sex chromosomes or polygenic sex determination [5], such as XX/XY [6,7,8], ZW/ZZ [9,10], XX/XY1Y2 [11], X1X2Y [12], X1X1X2X2/X1X2Y [13,14], XX/XO and ZO/ZZ [15].

Sexual dimorphism in traits such as growth rate, maturity age, body size, and color pattern is highly significant in many cultured fish species and has direct implications for production performance [16,17]. Sex control in fish is an effective breeding strategy that allows for more efficient and sustainable fish farming, enhancing yield and economic outcomes, especially when employing mono-sex population production strategies [18,19,20,21]. The development and application of sex-linked molecular markers have become invaluable tools in aquaculture, facilitating the production of mono-sex populations, early sex identification, and the enhancement of sex-linked traits, all of which are increasingly being applied in aquaculture [22,23,24,25,26,27,28]. In certain farmed fish species, males exhibit faster growth rates than females, as observed in Nile tilapia (*Oreochromis niloticus*) [29], channel catfish (*Ictalurus punctatus*) [30], yellow catfish (*Pelteobagrus fulvidraco*) [31,32], and Lanzhou catfish (*Silurus lanzhouensis*) [33]. Conversely, in other farmed species, females outgrow males, such as the half-smooth tongue sole (*Cynoglossus semilaevis*) [34], rainbow trout (*Oncorhynchus mykiss*) [35], sea bass (*Dicentrarchus labrax*) [36], Europeaneel (*Anguilla anguilla* L.) [37], and Southern catfish (*Silurus meridionalis*) [38]. Understanding the genetic architecture of sex determination in aquaculture species is essential for improving the efficiency, sustainability, and precision of breeding programs [39]. By integrating these genetic insights into practical applications, production efficiency will not only be improved but also reliance on older, less sustainable methods like hormonal treatments will be reduced, leading to a more technologically advanced and ethical aquaculture industry. Therefore, research on the genetic mechanisms underlying sex determination holds significant value for the execution of fish production strategies and sex control breeding [40].

Developing and utilizing sex-specific markers in fish breeding is a game-changing advancement for aquaculture, allowing for more precise control over the sex of populations, leading to increased productivity, efficiency, and sustainability [2]. The development of sex-specific markers has significantly advanced aquaculture breeding over the past two decades, leading to more efficient and sustainable production systems [17]. Traditional techniques such as Restriction Fragment Length Polymorphism (RFLP), Amplified Fragment Length Polymorphism (AFLP), Random Amplified Polymorphic DNA (RAPD), and Simple Sequence Repeat (SSR) have been crucial in the identification of sex-specific markers in over 20 aquaculture species [16,17]. However, these methods are now considered to be time-consuming, labor-intensive, and less effective compared to modern genomic techniques. Based on next generation sequencing (NGS), SNP markers have become a cornerstone of modern aquaculture breeding programs, enabling more efficient, precise, and sustainable practices [17,41]. By facilitating early selection for desired traits, improving disease resistance, enhancing feed efficiency, and maintaining genetic diversity, SNP markers allow breeders to meet the growing demand for seafood in an environmentally and economically sustainable manner. For example, restriction site-associated DNA sequencing (RAD-seq) [42,43], Type IIB Restriction Site-Associated DNA Sequencing (2b-RAD) sequencing [24,44], and whole-genome resequencing (GWS) [45,46,47] have been used to identify sex-specific markers. Recently, whole-genome sequencing technology has been increasingly applied for sex-specific marker screening in a growing number of farmed fish species [48,49], including largemouth bass (*Micropterus Salmoides* L.) [47], blunt-snout bream (*Megalobrama amblycephala*) [46], army fish (*Spinibarbus hollandi*) [45], *Spinibarbus hollandi* [23], *Takifugu bimaculatus* [49], blue tilapia (*Oreochromis aureus*) [50], yellowstripe goby (*Mugilogobius chulae*) [51], Russian sturgeon (*Acipenser gueldenstaedtii*) [26], turbot (*Scophthalmus maximus*) [52], and spotted knifejaw (*Oplegnathus punctatus*) [12].

The Northern snakehead (*Channa argus*) is a renowned aquaculture species with significant economic value. According to the China Fishery Statistical Yearbook, national aquaculture production of Northern snakehead surpassed 605,438 tons in 2023. Male individuals exhibit a markedly faster growth rate than females, particularly after the first year, with males reaching an average weight of more than double that of females. Due to this rapid growth, males are favored, growing nearly twice as fast as females. Consequently, all-male mono-sex breeding could substantially enhance breeding yield and economic benefits in Northern snakehead aquacultures [20,53,54]. Understanding the genetic and environmental mechanisms governing sex regulation and differentiation in Northern snakeheads, aquaculture operations can develop single-sex breeding strategies to optimize growth, improve yield, and control reproduction. A rapid and accurate sex-specific marker is indispensable for aquaculture breeding programs focused on producing all-male populations and identifying sex-reversed individuals. Previous research has developed several sex markers and fine-mapped the sex quantitative trait locus (QTL) [53,55,56,57]. However, the lack of understanding of sex chromosomes and the mechanistic action of the sex-determining genomic region presents a major obstacle in the development of efficient sex-specific markers for identifying YY super-males. In this study, we aimed to identify the sex determination region and putative sex-determining genes, as well as to develop genetic sex markers for the Northern snakehead. We conducted whole-genome resequencing on 59 Northern snakehead individuals. The GWAS results revealed a specific sex-determining chromosomal region on Chr03. By integrating the GWAS and RNA-seq results, we identified key candidate sex-determining genes. Moreover, two sex-specific InDel markers were validated for practical verification of genetic sex in XX, XY, and YY individuals, confirming an XX/XY sex determination system in Northern snakeheads. This effort will not only contribute to the theoretical understanding of sex differentiation but also provide practical tools for aquaculture, enabling more efficient and sustainable breeding strategies in regard to Northern snakeheads.

## 2. Results

### 2.1. Resequencing, Variants Calling and Population Structure Analyses

**Figure 1:**
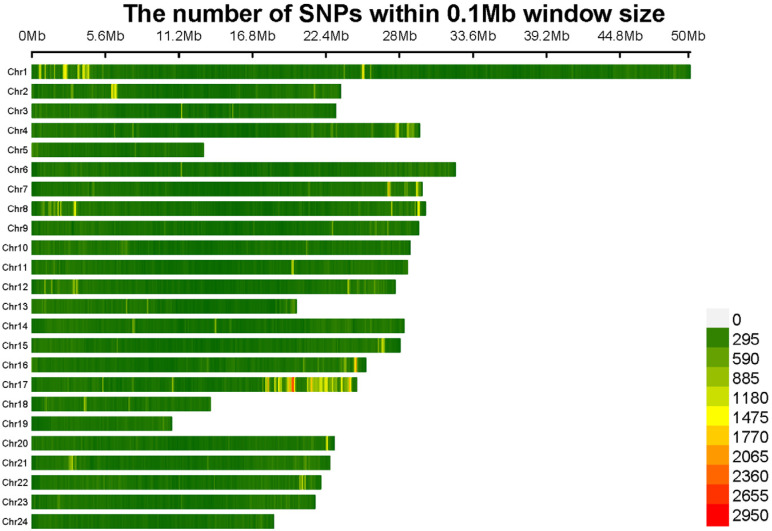
High-quality SNPs were mapped to 24 *C. argus* chromosomes, with the gradient color ranging from green to red to indicate increasing SNP density at 1 Mb intervals.

The genotyping of 59 adult Northern snakeheads was conducted using whole-genome resequencing. Approximately 871.3 Gb of raw data were generated using the Illumina NovaSeq platform. Following quality control, 864.3 Gb of clean reads were obtained. For the 59 individuals, the average amount of clean data per sample was 14.6 Gb. Based on the genome size, the mean alignment rate was 98.5% and the mean sequencing depth was 21.4×. A total of 4,613,089 SNPs and 834,465 InDels were identified using HaplotypeCaller in GATK. Following additional quality control using Plink v. 1.90, which included filtering by a missing call rate and minor allele frequency (MAF), the high-quality site of 1,087,459 SNPs and 159,324 InDels were retained for subsequent GWAS. The SNP density map in Figure 1 showed that the number of SNPs per chromosome ranged from 11,125 on chromosome 19, which had the fewest SNPs, to 50,096 on chromosome 1, which had the most. SNPs were uniformly distributed across the chromosomes, with the average density exceeding 7318 loci per Mb.

### 2.2. Sex-Linked Region and Candidate Gene of C. argus

**Figure 2:**
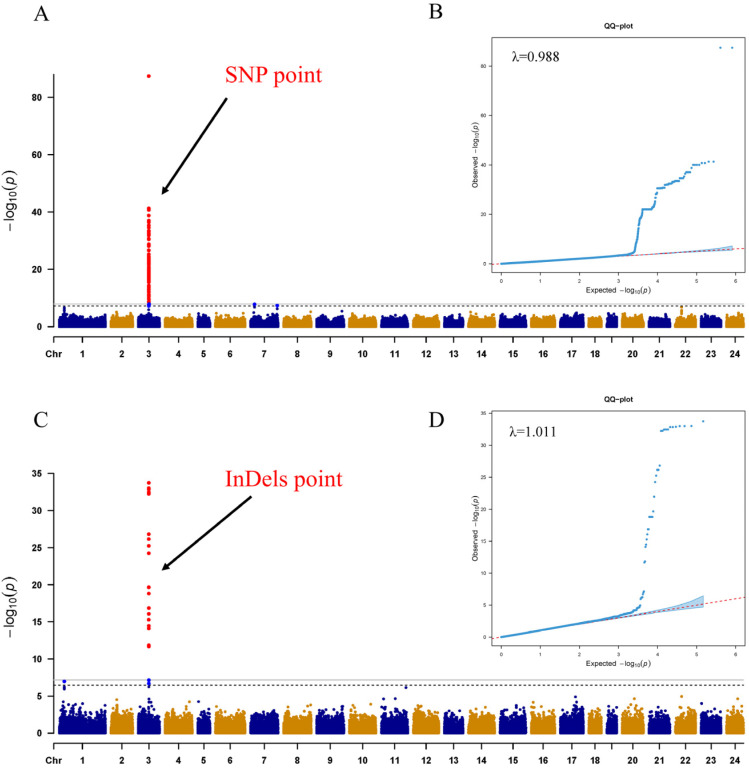
Results of GWAS analyzing sex characteristics. (**A**,**C**) show Manhattan plots of the optimal patterns of SNPs and InDels, along with Q-Q plots for SNPs, InDels, and corresponding λ values. The *x*-axis represents the chromosome number (Chr) while the *y*-axis represents the −log10(P) value for each SNP. Red dots indicate SNP signals exceeding the significance threshold, whereas blue and yellow dots fall below the threshold. The red points indicated by the black arrows represent significant loci. (**B**,**D**) present Q-Q plots of the optimal patterns of SNPs and InDels.

A total of 270 SNPs were found to be significantly associated with sex on chromosome 03 (Chr 03) (Figure 2A, Table S1). These SNPs accounted for 3.97% to 98.97% of the phenotypic variance explained (PVE). Similarly, 31 InDels were identified as significantly associated with sex on Chr 03 (Figure 2C, Table S1). These InDels accounted for 7.30% to 42.61% of the PVE. The Q-Q plots and lambda (λ) values confirm that the GWAS model used is well calibrated and appropriate for analyzing the SNP and InDel data (Figure 2B,D).

**Figure 3:**
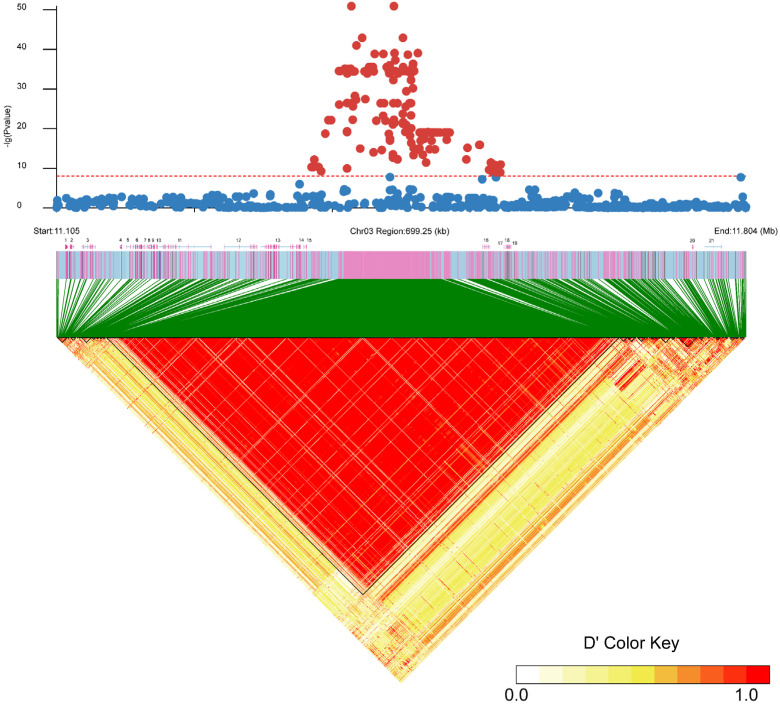
Fine mapping on Chr 03 revealed the association between SNPs and sex as well as the combined linkage disequilibrium in the sex-related region. Red dots indicate SNP signals exceeding the significance threshold, whereas blue dots fall below the threshold. The axis below the chart represents the region where candidate genes are annotated.

All SNPs were clustered within a 192 Kb segment on Chr 03 (11,363,773 bp to 11,555,658 bp) (physical location of the start and end in the reference genome). Although two uncharacterized proteins and the *rnf144a* protein-encoding gene were identified among the 270 SNPs, their lack of involvement highlights the functions of sex determination or differentiation. Additionally, 31 InDels were significantly associated with sex within a 143 Kb region on Chr 03 (11,390,044 bp to 11,534,257 bp), with many loci conforming to the XX/XY pattern. Furthermore, the identification of 21 potential genes through scanning the ±250 Kb regions around genome-wide significant variants, shown in linkage disequilibrium (LD) blocks (Figure 3), is annotated in Table 1. The genes closest to the sex-determining region are EVM0022341 (uncharacterized protein), EVM0015303 (Ring finger protein), and EVM0022102 (uncharacterized protein), located at Table 1. Interestingly, *Sox11* and *Id2* were located downstream and upstream of the sex-determining region.

### 2.3. Selection and Test of the Sex-Specific Markers

**Figure 4:**
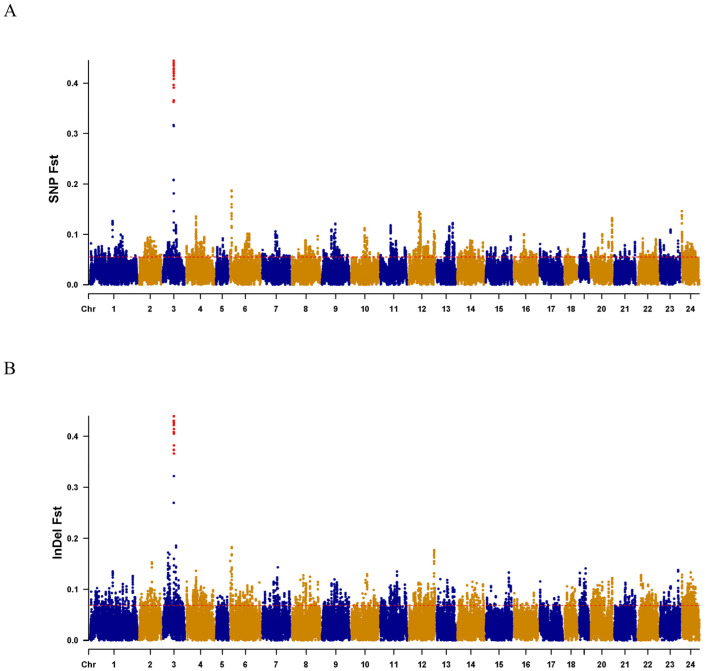
Result of Fst scan for females and males on chromosomes. (**A**,**B**), fine mapping on Chr 3 shows the combination of associations of SNPs and InDels with sex and Fst, respectively. Candidate sign inferred by Fst (>0.3490) are marked by red dots, whereas blue and yellow dots fall below the Fst (>0.3490). The *x*-axis indicates the Chr numbers. The *y*-axis is the Fst value of the female and male of the SNPs or InDels.

**Figure 5:**
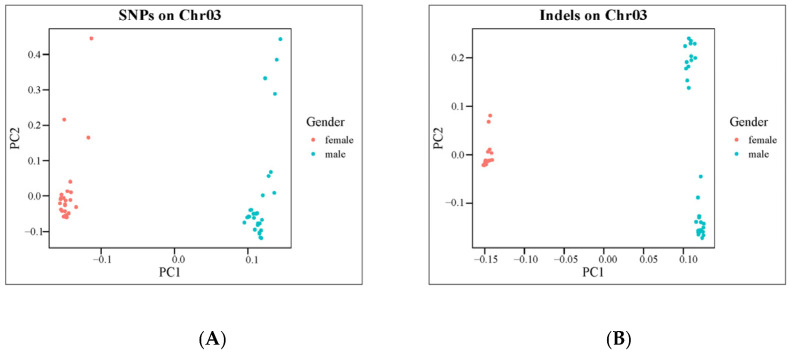
Result of PCA for females and males with traits. (**A**,**B**), the PCA of 59 individuals to distinguish two sexes using SNPs or InDels on Chr03. The red and blue dots represent females and males, respectively.

**Figure 6:**
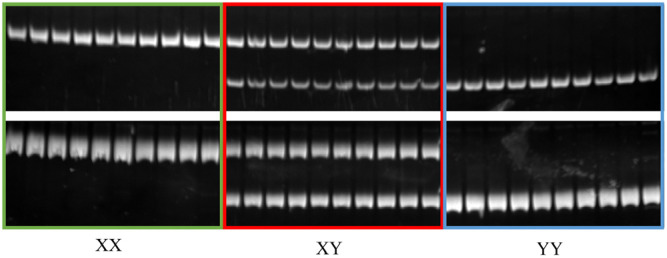
Results of PCR products for females, males, and super-males with two primers. The band above the green frame denotes female samples (XX), the red frame represents male samples (XY), and the blue frame signifies super-male samples (YY) within the Northern snakehead population.

Fst values (Figure 4A,B) and PCA analysis (Figure 5A,B) for each SNP and InDel between males and females confirmed the effectiveness of the primer design site. Subsequently, 10 pairs of male-specific primers were designed by screening 10 sexually dimorphic InDels from 31 identified InDels. After the preliminary PCR amplification screening, we successfully obtained two pairs of primers which effectively distinguished the *C. argus* of XX-F (genetic sex is XX and phenotypic sex is female), XY-M (genetic sex is XY and phenotypic sex is male), and YY-M (genetic sex is YY and phenotypic sex is male) (Figure 6). For both primers, all samples exhibited clear bands: males displayed two bands, while females and super-males showed a single band, located above and below, respectively.

### 2.4. Transcriptome Analysis

**Figure 7:**
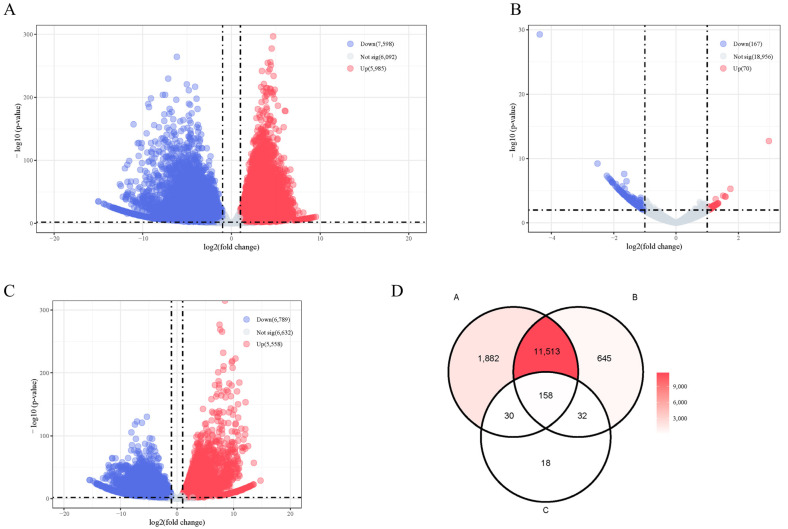
Transcriptome analyses based on gonads in Northern snakeheads. (**A**–**C**) Volcano plot of DEGs shows all the individuals’ gonads within RNA-seq. Red and blue dots indicate significantly upregulated and downregulated genes, respectively (|log2FC| ≥ 1 and *p* < 0.01). Gray dots indicate genes not significantly differentially expressed between XX vs. XY, XY vs. YY, and XX vs. YY. (**D**) Venn maps of DEGs obtained from RNA-seq based on gonads, whereas A is XX vs. XY, B is. XY vs. YY, and C is XX vs. YY. The overlapping intersection is compared with the RNA-seq result, and the resulting region of 158 represents the final conservative DEGs.

**Figure 8:**
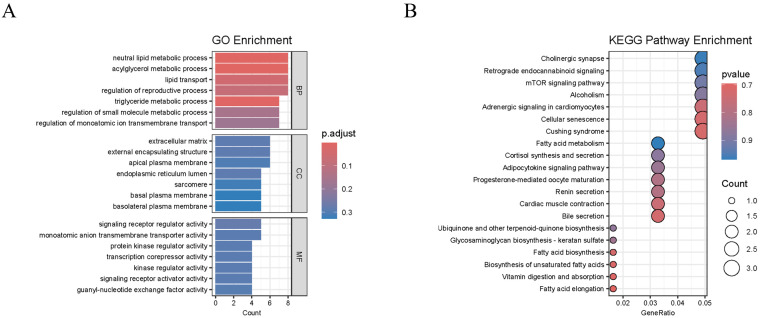
Functional enrichment analysis of overlapping DEGs. (**A**) presents the top 21 significantly enriched Gene Ontology (GO) terms for the 158 conserved DEGs. (**B**) shows the 20 significantly enriched KEGG pathways for the 158 conserved DEGs.

A total of 10,672 significant DEGs were identified in XY-M vs. XX-F, 237 were identified in XY-M vs. YY-M, and 12,347 were identified in YY-M vs. XX-F using RNA-Seq. In XY-M vs. XX-F, 5985 genes were upregulated and 7598 were downregulated; in XY-M vs. YY-M, 70 genes were upregulated and 167 were downregulated; in YY-M vs. XX-F, 5558 genes were upregulated and 6789 were downregulated (Figure 7A–C). A total of 158 conserved DEGs in representative glandular tissues were identified by overlapping the differentially expressed genes between groups using Venn diagrams (Figure 7D). These 158 DEGs were collectively enriched in 384 significant GO terms. Figure 8A shows the top GO enrichment, where biological pathways related to signal transduction regulation were significantly enriched, including neutral lipid metabolic process, lipid transport, regulation of monoatomic ion transmembrane transport, signaling receptor regulator activity, monoatomic anion transmembrane transporter activity, protein kinase regulator activity, transcription corepressor activity, and kinase regulator activity. In the KEGG enrichment analysis (Figure 8B), 20 pathways were significantly enriched. Fatty Acid Elongation, Fatty Acid Metabolism, and Fatty Acid Biosynthesis are important for the growth and function of gonadal cells and influence the production of sex hormones. The biosynthesis of Unsaturated Fatty Acids and the Adipocytokine Signaling Pathway play pivotal roles in energy metabolism and reproductive function, regulating gonadal activity and the secretion of sex hormones. Furthermore, the Progesterone-mediated Oocyte Maturation (POM) and mammalian target of rapamycin (mTOR) Signaling Pathways are of particular importance, with POM being directly involved in ovarian function, regulating the process of oocyte maturation and ovulation. The mTOR pathway is critical for cell proliferation and metabolic regulation, significantly impacting gonadal development and germ cell production.

**Figure 9:**
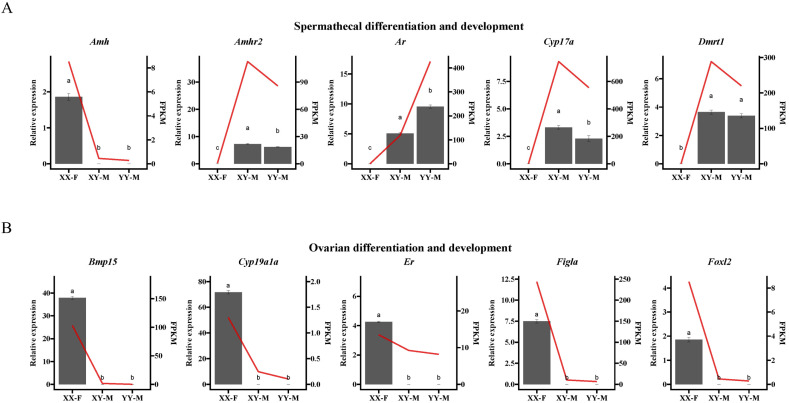
qPCR validation for (**A**) five genes within the spermathecal differentiation and development DEGs of gonads RNA-seq and (**B**) five genes within the ovarian differentiation and development DEGs of gonads RNA-seq. The gray bars represent the relative expression levels of the gene, corresponding to the *y*-axis on the left side of each figure, and are presented as mean ± standard deviation. The red line indicates the gene expression levels from RNA-seq data, represented in FPKM, and corresponds to the *y*-axis on the right side of each figure, displayed as the mean. The significance of the qPCR results between the XX-F, XY-M, and YY-M individuals is indicated by letters. The *x* axis that XX-F (genetic sex is XX and phenotypic sex is female), XY-M (genetic sex is XY and phenotypic sex is male) and YY-M (genetic sex is YY and phenotypic sex is male).

The qPCR results showed that *Amh*, *Amhr2*, *Ar*, *Cyp17a*, and *Dmrt1* were significantly more highly expressed in XY-M and YY-M spermatheca compared to XX-F (Figure 9A), while the ovarian differentiation-related genes *Bmp15*, *Cyp19a1a*, *Er*, *Figla*, and *Foxl2* were significantly under expressed in XY-M and YY-M spermatheca (Figure 9B). These results are consistent with the expression patterns observed in the transcriptomic analysis, which demonstrated clear sexual dimorphism, although the relative expression levels varied slightly. Further correlation analysis was conducted between the qRT-PCR results of DEGs related to spermathecal and ovarian differentiation and the transcriptomic data. Significant correlations were observed for the XY-M vs. XX-F and XY-M vs. YY-M groups, with R² values of 0.8835 and 0.9008, respectively, indicating the high reliability of the transcriptomic data.

### 2.5. Identification of Candidate Genes by GWAS and RNA-Seq

**Figure 10:**
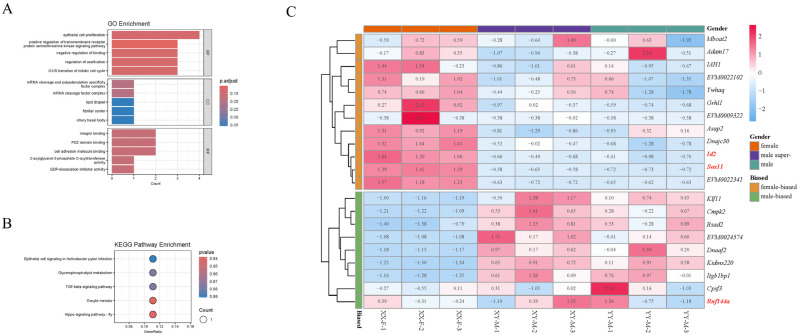
Final candidate genes and their functional enrichment analysis. (**A**,**B**) Enriched GO terms and KEGG pathways for candidate genes. (**C**) Heatmap analysis of candidate genes differentially expressed between XX-F, XY-M and YY-M Northern snakeheads. XX-F, female gonad individuals. XY-M, male gonad individuals. YY-M, super-male gonad individuals. Matrix blocks in red are upregulated while those in blue are downregulated in gens. The red and bold name is interested in candidate gene.

A total of 21 potential genes were extracted and annotated in the 250 kb gene candidate region near the locus of significance SNPs. Also, to identify sex-biased genes within the candidate sex-determining regions, we compared these genes with the DEGs in the RNA-Seq dataset of sexually active gonads. The results showed that it contained 10 female-biased transcripts and seven male-biased transcripts (Figure 10B, Table S2). The female-biased protein-coding genes identified were *ywhaq*, *iah1*, *asap2*, *id2*, *sox11*, *dnajc30* and *grhl1*. The male-biased genes included *klf11*, *cmpk2*, *rsad2*, *dnaaf2*, *itgb1bp1*, and *kidins220*, along with other uncharacterized genes. The finding of these genes will assist in the foundation of future in-depth studies of the molecular mechanisms of Northern snakehead populations. We conducted a functional exploration of candidate genes by GO enrichment and a Kegg pathway enrichment analysis (Figure 10A,C). The results revealed that several GO enrichment terms might be associated with sex differentiation or development, including DP-dissociation Inhibitor Activity, Lipid Droplet, Positive Regulation of Transmembrane Receptor Protein Serine/Threonine Kinase Signaling Pathways, 2-acylglycerol-3-phosphate O-acyltransferase Activity and mRNA Cleavage Factor Complexes and mRNA Cleavage and Polyadenylation Specificity Factor Complexes. Additionally, five KEGG pathways were significantly enriched among these candidate genes, with Hippo Signaling Pathway—Fly, Oocyte Meiosis, TGF-beta Signaling Pathway, and Glycerophospholipid Metabolism being potentially associated with sex regulation.

## 3. Discussion

In recent years, WGS has become a powerful tool in the development of sex-specific markers due to its broad coverage, ability to detect large sex-linked fragments, and capability of screening vast numbers of genetic markers, and it has been increasingly applied to the development of sex-specific markers in a growing number of fish species [45,46,49]. Based on resequencing, several new analytical strategies have been developed for sex-specific sequence screening, including the investigation of sexually dimorphic SNPs and the comparing of sequencing depth, GWAS, and Fst analyses [10,58,59]. We conducted GWAS on sex traits in Northern snakeheads and identified a total of 270 SNPs and 31 InDels associated with the sex phenotype. All these phenotype-associated SNPs and InDels exceeded the significance threshold. Among the genes associated with these molecular markers, three were associated with SNPs, two with InDels, and two genes were found to be associated with both SNPs and InDels. Subsequent studies have shown that the genes EVM0022341 and *rnf144a* are not directly related to sex determination or differentiation functions. Interestingly, *sox11* and *id2* were found within their sex determination candidate window.

Clarifying the sex determination mechanism in Northern snakeheads, including the confirmation of its XX/XY system and the identification of a sex-determining region through QTL mapping, lays the groundwork for mono-sex breeding strategies [53,55,56,57]. The development of sex-specific markers using AFLP [60] and SSR [61] methods presents several disadvantages: they are costly, labor-intensive, and time-consuming. First, while SSR markers have historically been useful for identifying sex-specific markers, the process of isolating and screening them is time-consuming and labor-intensive. SNP, however, is a powerful, versatile, and efficient tool that can better facilitate the identification and utilization of snakeheads [62]. The use of specific primers designed from GWAS results for PCR validation is a key step in confirming the sex-specificity of identified genetic fragments to ensure the accuracy of sex-linked markers and their applicability in breeding programs, which ultimately contributes to more efficient and precise sex control in aquaculture species. Based on 31 sex-related InDels, 10 pairs of primers were designed. Finally, two InDel sex-linked markers were obtained that can successfully distinguish XX, XY, and YY fish. The results suggest that this method offers advantages in terms of precision and lower sample size requirements. This method has been gradually applied to assist in mapping sex-specific sequences in aquatic animals, such as largemouth bass (*Micropterus Salmoides* L.) [47], blunt-snout bream (*Megalobrama amblycephala*) [46], army fish (*Spinibarbus hollandi*) [45], *Spinibarbus hollandi* [23], *Takifugu bimaculatus* [49], Pacific abalone (*Haliotis discus*) [48], and *Takifugu bimaculatus* [49]. Sun-developed universal sex markers (*C. maculata* ♀ × *C. argus* ♂) are applicable to two species, *C. argus* and *C. maculata*, and their hybrids [56]. Although these markers are effective in distinguishing genetic males and females in snakehead species, they cannot distinguish YY fish. Additionally, Dai [63] have developed a molecular marker for sex identification in albino snakeheads which can differentiate YY fish from albino snakeheads and also distinguish between male and female wild snakeheads. However, there is no direct evidence for distinguishing XX, XY, and YY fish from non-albino snakeheads. Sex markers in these species usually offer technical advantages. Wen employed a single analytical method to display the Fst values between males and females across the entire genome, identifying a substantial potential sex differentiation region spanning 6 Mb and encompassing 275 genes, representing a relatively broad research scope [46]. In the present study, we used both GWAS and Fst to identify sex-linked genomic segments. A total of 270 SNPs and 31 InDels were detected as sexually dimorphic regions spanning a narrow 115 Kb segment on Chr03, providing a valuable resource for developing diagnostic markers of genetic sex in Northern snakeheads. In general, these new sex markers establish a strong foundation for identifying the genetic sex and sex chromosomes of Northern snakeheads and provide a highly efficient technological approach for mono-sex breeding in Northern snakehead aquaculture, which holds significant potential for enhancing efficiency and productivity.

Among the numerous sex-specific markers identified in fish, some are located on sex-determining genes. For example, the domain gene on the Y chromosome (DMY) gene was found in the sex-determining region of Medaka (*Oryzias latipes*) [64], the *dmrt1* gene was identified based on a male-specific 15 bp deletion in large yellow croaker (*Larimichthys crocea*) [65], the *dmrt1* gene in spotted scat (*Scatophagus argus*) [66], and the *amh* gene in mandarin fish (*Siniperca chuatsi*) [67]. However, in some fish species, sex-determining genes have not been identified despite the presence of sex markers, such as in spotted knifejaw [68], blotched snakehead [69], blunt-snout bream (*Megalobrama amblycephala*) [46], albino snakeheads [63] and largemouth bass [51]. In our previous study, several genes, including *sox11*, were identified in the sex QTL region using a high-density genetic map and QTL analysis [53]. In this study, based on a high-quality reference genome and GWS combined with GWAS analysis, we accurately identified the sex-determining region on Chr 03 within a 115 Kb segment, facilitating the screening of candidate genes. However, no previously identified sex-determination genes were found in the sex-determining region based on the annotation results. The genes closest to the sex-determining region are EVM0022341 (uncharacterized protein), EVM0015303 (Ring finger protein), and EVM0022102 (uncharacterized protein). Additionally, *sox11* and *id2* were near the window of sexual differentiation.

Recently, fish have been observed to exhibit sex plasticity, displaying a diverse array of sex determination mechanisms. As high-throughput sequencing technology has become more sophisticated, numerous candidate genes have been identified across various teleost fish species. For example, the amh in Sebastes rockfish [70], the *dmrt1* in Scatophagus argus [66], the *sox11a* gene in *Oreochromis niloticus* [71], and five sex-related genes in largemouth bass [72]. In this study, the *sox11* protein-coding gene was annotated from the ~115 Kb candidate SD region on Chr 03. Sox11 is a transcriptional activator that belongs to the SOX gene family and is crucial for several developmental processes, including gonadal differentiation, spermatogenesis, vitellogenesis, and oogenesis in fish [71,73,74]. This gene corresponds to the candidate gene *sox11* identified in the fine mapping of the sex QTL [53]. Additionally, RNA-Seq analysis revealed that the *sox11* gene is the most highly expressed in females among the three gonadal tissues, indicating that the *sox11* gene may play a complex role in the sexual differentiation of Northern snakeheads. Similar results were obtained in *Collichthys lucidus* [75].

To date, several new candidate master sex-determining (MSD) genes have been discovered that are not directly related to gonadal development. For example, the Y-chromosome (*sdY*) gene in Atlantic Salmon [76,77,78,79], the *bcar1* gene in channel catfish [80], the *rnf183* gene in *L. polyactis* [81], and the *id2bbY* gene in *Arapaima gigas* [82], Interestingly, we characterized two protein-coding genes, *rnf144a* and *id2*, consistent with findings from a recent study [56], in candidate sex-determining regions that have not yet been functionally confirmed. *id2* proteins belong to the ID family and primarily regulate cell differentiation and proliferation by inhibiting the function of bHLH (basic Helix–Loop–Helix) transcription factors. Evidence has demonstrated that the *id2* gene plays a crucial role in male meiosis and spermatid development in mice by regulating the differentiation of testicular supporting cells through the inhibition of bHLH transcription factors [83]. The *id2bbY* gene, a homolog of *id2* in *Arapaima gigas*, is thought to be involved in sex determination [82]. *rnf144a*, an E3 ubiquitin ligase for DNA-PKcs, promotes apoptosis during DNA damage and is strongly correlated with body conformation traits in goats [84]. Although it has rarely been reported in teleost fish, a recent study on RNF family members showed that *rnf183* is a sex-determining gene in little yellow croaker [81]. In our study, the *id2* genes showed biased expression in both females and males in RNA-seq, whereas *rnf144a* exhibited inconsistent expression trends. Therefore, it is reasonable to assume that the *id2* or *rnf144a* gene might be a candidate MSD gene in *C. argus*. These genes represent valuable genetic resources for studying the sexual evolution of the Northern snakehead and other teleost fish. By focusing on these genes, researchers can gain insights into the origin and evolution of primitive sex chromosomes, the genetic mechanisms driving sex determination, and how these systems have evolved over time. This knowledge has important implications for both evolutionary biology and practical applications in aquaculture, particularly in the development of sustainable and efficient mono-sex breeding strategies.

By combining the RNA-seq data from three gonadal tissues of *C. argus*, the candidate genes *id2* and *rnf144a* were analyzed in the sex determination region, and it was found that they exhibited biased expression related to sex determination in *C. argus*. This finding warrants further investigation, particularly focusing on the roles and regulatory mechanisms within the sex determination (SD) system. By employing gene-editing technologies and gene expression profiling in gonads during the critical stages of sex determination, we can uncover the precise roles of the master sex-determining genes in Northern snakeheads in future works. This will not only enhance our understanding of sex differentiation but also pave the way for developing targeted genetic tools to control sex ratios in aquaculture, leading to more sustainable and productive breeding programs.

## 4. Materials and Methods

### 4.1. Ethics Statement

The sampling process of *Channa argus* adhered to the experimental animal welfare guidelines and was approved by the Experimental Animal Welfare Ethics Committee of the Pearl River Fisheries Research Institute, Chinese Academy of Fishery Sciences.

### 4.2. Sample Sources and DNA Extraction

**Figure 11:**
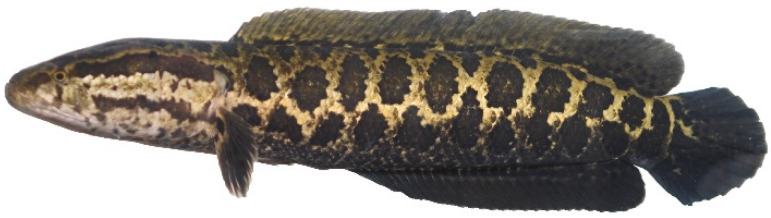
Individuals in *C. argus*.

**Figure 12:**
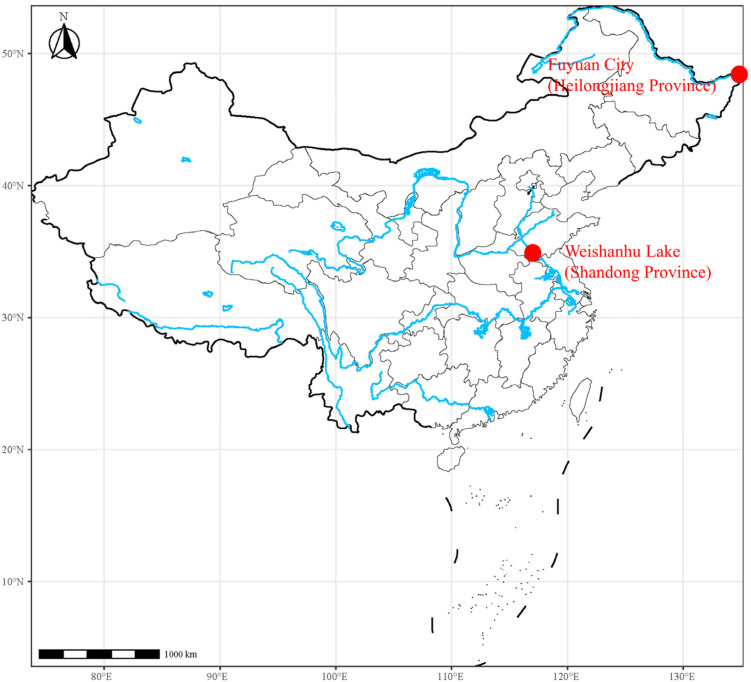
Map of sampling sites for *C. argus*. The red dot shows the specific sampling locations.

Adult Northern snakehead (Figure 11) samples were originally collected from Weishanhu Lake (Shandong Province) and Fuyuan City (Heilongjiang Province) in China (Figure 12). These fish were cultivated at the Nanhai Bai-rong Aquatic Varieties Limited Company in Foshan City, Guangdong Province, China. The phenotypic sex of all individuals was determined by examining the gonads. A total of 59 fish (26 females and 33 males) were randomly selected, and fin tissue was collected from each individual, immediately immersed in absolute ethanol, and then preserved at −20 °C. Genomic DNA was extracted from the fin clips using a Qiagen DNeasy Blood and Tissue Kit (Qiagen, Valencia, CA, USA) according to the manufacturer’s protocol. DNA quality and concentration were assessed using a NanoDrop One spectrophotometer (Thermo Scientific, 168 Third Avenue, Waltham, MA, USA) and 1% agarose gel electrophoresis.

### 4.3. Whole Genome Resequencing and Variation Detection

Genomic DNA from 59 individuals was used for whole-genome resequencing, which was performed on the Illumina NovaSeq platform in PE150 mode. The raw reads obtained from the high-throughput sequencing pipelines were quality controlled using FastQC software (http://www.bioinformatics.babraham.ac.uk/projects/fastqc/, accessed on 6 October 2024). Paired-end data with adapter contamination and low-quality bases were trimmed to obtain clean reads using fastp (v. 0.21.0) with default parameters [85]. The clean reads were aligned to the Northern snakehead reference genome (ASM1899790v1) using BWA (v. 0.7.17) [86]. The resulting SAM files were converted to BAM files and sorted using SAMtools (v1.15.1) [87]. Duplicates were removed with the MarkDuplicates tool from the Picard toolkit v2.27.5. SNPs and InDels were identified using the Genome Analysis Toolkit (GATK) package (v. 4.3.0.0), following handle scripts, including the merging of each sample gVCF files and combining them into a multi-sample gVCF file by HaplotypeCaller and CombineGVCFs tools; Genotyping multi-sample gVCF file with GenotypeGVCFs methods, finally the SNP and InDel after filtering the variants following the parameters “QD < 2.0 || MQ < 40.0 || FS > 60.0 || SOR > 3.0 || MQRankSum < −12.5 || ReadPosRankSum < −8.0”. The resulting SNPs and InDels were further filtered using PLINK (v. 1.90) [88], excluding sites with missingness per individual > 0.1 and missingness per marker > 0.05.

### 4.4. Sex-Determining Region and Loci Screening Using GWAS

A genome-wide association analysis of the sex phenotype data and genotype data was conducted using a mixed linear model (MLM) in GEMMA (v. 0.98.5) software, based on the filtered SNPs and InDels [89]. The MLM is represented by the following equation: Y=Xβ+Zμ+e. Here, *Y* represents the observation vector of phenotypes, *X* is the design matrix of fixed effects, including the first three principal components and sex, and *β* is the vector of fixed-effect coefficients, often used to explain global, systemic impacts. *Z* is the design matrix of random effects, *μ* is the vector of random-effect coefficients (reflecting random differences between individuals as well as genetic background or population structures), and *e* represents the residual effects [90].

In the present study, we used the phenotypic characteristics of males and females as a basis for identifying genes in sex-determining regions. To ensure the validity of the optimal GWAS mode analysis, we first used a genomic inflation factor (λ) value close to 1.00. [91]. Quantile-quantile (Q-Q) plots and a Manhattan plot of significant SNPs or InDels were visualized by CMplot [92]. The genome-wide significance and suggestive association thresholds were set at 0.01/N and 0.05/N, respectively, where N represents the number of SNPs and InDels used in the GWAS analysis. These thresholds were established according to the Bonferroni correction method [93].

### 4.5. Sex Candidate Gene and Region Identify by Fst

The sex-linked genomic segment in Northern snakeheads was pinpointed through GWAS utilizing filtered SNPs. The Fst [94] between females and males was also calculated at each SNP, and the results were plotted using a CMplot to investigate sex-linked genomic regions. The significance of the association between loci and phenotypic sex was determined using the resulting threshold values, which were then plotted to further examine sex-linked genomic regions. To identify candidate genes, LDBlockShow (v. 1.40) was employed to generate linkage disequilibrium (LD) blocks and construct haplotype maps for the 250 kb regions upstream and downstream of each significantly associated SNP [95]. Subsequently, candidate genes within the delineated haplotype block regions were precisely annotated using BLAST against the SwissProt protein database.

### 4.6. Male-Specific Markers Development and Validation through InDel Region

We used a script to identify samples with sexually dimorphic InDels sites larger than 30 bp, and their validation was performed by aligning the resequencing reads from female and male samples to the reference genome. The selected data were identified as candidate regions using IGV (http://www.broadinstitute.org/software/igv/, accessed on 6 October 2024) for following PCR primers (Tables S3 and S4). These primers were designed based on the genomic sequences using Primer-BLAST (https://www.ncbi.nlm.nih.gov/tools/primer-blast/index.cgi, accessed on 6 October 2024), and these primers were then synthesized by Tianyi Huiyuan Biotech Co., Ltd. (Guangzhou, China). To verify the specificity of the primers, routine PCR amplification was conducted using the primer pairs synthesized in bulk by the aforementioned company. The PCR reaction was conducted in a total volume of 20 μL, consisting of 10 μL of Taq Mix, 0.5 μL of DNA template, 0.8 μL of each forward and reverse primer, and 7.9 μL of deionized water. The PCR conditions included an initial denaturation at 95 °C for 2 min, followed by 39 cycles of 35 s at 95 °C for denaturation, 35 s for annealing at the Tm, and 40 s at 72 °C for extension, with a final extension at 72 °C for 10 min. The amplified product length was evaluated by separate 8% polyacrylamide gels on 100 samples, including 43 females and 57 males, from randomly selected groups.

### 4.7. RNA Extraction, Sequencing, and Analysis of Sex-Biased Genes

RNA was extracted from the gonads of three Channa argus genotypes (XY-M, XX-F, and YY-M) using TRIzol reagent (Invitrogen, Carlsbad, CA, USA). The quality and concentration of RNA from each sample were assessed before being sent to Beijing Novogene (Beijing, China) for library construction and paired-end sequencing using the Illumina HiSeq 2000 platform (Illumina, San Diego, CA, USA). All raw sequencing data have been deposited in the NCBI database under accession number PRJNA895982.

Raw data were processed using FastQC (v. 0.11.8) to remove adapters, bad-quality reads, and reads that carried ambiguous bases (N), resulting in high-quality clean data. The clean reads were then aligned to the Northern snakehead reference genome (GenBank assembly accession No. GCA_003845795.1) using HISAT2 (v. 2.1.0) [96]. Transcript expression levels were quantified using the fragments per kilobase of transcript per million mapped reads (FPKM) method [97]. Differential expression analysis between sample groups was performed using DESeq2 (v. 1.36.0) to identify differentially expressed genes (DEGs) [98]. The false discovery rate (FDR) was controlled by adjusting *p*-values, with significance thresholds being set at |log2 fold change| ≥ 1 and *p* < 0.01 [98]. Gene Ontology (GO) and Kyoto Encyclopedia of Genes and Genomes (KEGG) pathway enrichment analyses were conducted using the ClusterProfiler package (v. 3.18.1) in R [99,100,101]. Candidate genes identified through GWAS were compared with the DEGs to identify overlapping genes. These overlapping genes were clustered based on Pearson’s correlation and Ward’s method using the R function hclust and subsequently visualized as heatmaps with the pheatmap package (v. 1.0.12) [102].

### 4.8. Validation of the Candidate Genes in RNA-Seq by qPCR

We designed primer pairs targeting 10 sex-related genes and employed quantitative PCR (qPCR) to validate the reliability of the RNA sequencing results. The reaction mixture (total volume of 20 μL) was prepared following the manufacturer’s instructions (ABI, Tampa, FL, USA) and included 10 μL of 2×SYBR Green^®^ Real-Time PCR Master Mix (Toyobo, Osaka, Japan), 0.8 μL of paired primers, 0.5 μL of cDNA template, and 7.9 μL of sterile water. The following PCR conditions were employed for amplification: three biological replicates were performed and then standardized using *C. argus* β-actin as an internal reference [103]. Relative gene expression levels were calculated using the 2^−ΔΔCt^ method [104]. One-way ANOVA was conducted using SPSS 22.0 to assess the significance of gene expression differences between groups, with *p* < 0.05 being considered statistically significant.

## 5. Conclusions

In the present study, we identified sexually linked gene fragments on Chr03 and developed two sex-specific markers. Additionally, we observed many growth-related candidate genes located near the associated SNPs. These findings provide a foundation and essential tools for further exploration of the molecular mechanisms of sex determination, ultimately facilitating advancements in the sex control breeding of Northern snakehead populations.

## Figures and Tables

**Table 1:** The candidate genes in the sex-determining region on Chr 03 for Northern snakehead.

ID	Gene Position	Description	Candidate Gene
1	11,113,901~11,116,316	Required for cytoplasmic pre-assembly of axonemal dyneins, thereby playing a central role in motility in cilia and flag	EVM0016285
2	11,117,840~11,123,073	KLF transcription factor 11	*Klf11*
3	11,130,447~11,144,557	Grainyhead-like protein 1 homolog	*Grhl1*
4	11,169,451~11,171,481	uncharacterized protein	EVM0024574
5	11,175,684~11,180,589	Tyrosine 3-monooxygenase tryptophan 5-monooxygenase activation protein, theta polypeptide b	*Ywhaq*
6	11,182,245~11,194,044	Disintegrin and metalloproteinase	*Adam17*
7	11,195,427~11,197,648	Isoamyl acetate-hydrolyzing esterase 1 homolog	*Iah1*
8	11,198,272~11,199,288	Homolog subfamily C member	*Dnajc30*
9	11,200,437~11,204,673	Cleavage and polyadenylation	*Cpsf3*
10	11,206,096~11,207,457	Integrin beta 1 binding protein 1	*Itgb1bp1*
11	11,208,746~11,262,323	Arf-GAP with SH3 domain, ANK repeat and PH domain-containing protein	*Asap2*
12	11,275,093~11,308,850	Membrane bound O-acyltransferase domain containing	*Mboat2*
13	11,312,296~11,352,898	Kinase D-interacting substrate of	*Kidins220*
14	11,355,614~11,356,323	Inhibitor of DNA binding 2, dominant Negative helix–loop–helix protein	*Id2*
15	11,356,961~11,358,844	Uncharacterized protein	EVM0022341
16	11,537,084~11,544,994	Ring finger protein	*Rnf144a*
17	11,558,889~11,559,263	Uncharacterized protein	EVM0022102
18	11,560,799~11,562,796	Radical S-adenosyl methionine	*Rsad2*
19	11,563,209~11,566,190	(UMP-CMP) kinase 2	*Cmpk2*
20	11,749,994~11,751,085	SRY (sex-determining region Y)-box	*Sox11*
21	11,762,407~11,780,316	Uncharacterized protein	EVM0009322

ID, the numbers correspond to the positions in Figure 3; gene position, physical location of the start and end of the gene in the reference genome.

## Data Availability

The raw data analyzed in this study can be downloaded from the National Center for Biotechnology Information (NCBI) databases (PRJNA895982).

## Supplementary Materials

The following supporting information can be downloaded at: https://www.mdpi.com/article/10.3390/ijms252010889/s1.
